# Sensitivity-Selectivity Trade-Offs in Surface Ionization Gas Detection

**DOI:** 10.3390/nano8121017

**Published:** 2018-12-06

**Authors:** Gerhard Müller, J. Daniel Prades, Angelika Hackner, Andrea Ponzoni, Elisabetta Comini, Giorgio Sberveglieri

**Affiliations:** 1 Department of Applied Sciences and Mechatronics, Munich University of Applied Sciences, D-80335 Munich, Germany; 2 MIND Group—Departament d’Engiyeria Electrònica i Biomèdica, Universitat de Barcelona, C Martí i Franquès, 108028 Barcelona, Spain; dprades@el.ub.edu; 3 IN^2^UB, Institut de Nanociència i Nanotecnologia de la Universitat de Barcelona, C Martí i Franquès, 108028 Barcelona, Spain; 4 AIRBUS Central R&T, D-81663 Munich, Germany; angelika.hackner@airbus.com; 5 National Institute of Optics (CNR-INO), National Research Council, Via Branze 45, 25123 Brescia, Italy; andrea.ponzoni@unibs.it; 6 Department of Information Engineering, University of Brescia, Via Valotti 9, 25133 Brescia, Italy; elisabetta.comini@unibs.it (E.C.); giorgio.sberveglieri@unibs.it (G.S.); 7 NANO SENSOR SYSTEMS SRL—Via Valotti 9, 25133 Brescia, Italy

**Keywords:** surface ionization, gas detection, sensitivity, selectivity, corona discharge, secondary electron emission

## Abstract

Surface ionization (SI) provides a simple, sensitive, and selective method for the detection of high-proton affinity substances, such as organic decay products, medical and illicit drugs as well as a range of other hazardous materials. Tests on different kinds of SI sensors showed that the sensitivity and selectivity of such devices is not only dependent on the stoichiometry and nanomorphology of the emitter materials, but also on the shape of the electrode configurations that are used to read out the SI signals. Whereas, in parallel-plate capacitor devices, different kinds of emitter materials exhibit a high level of amine-selectivity, MEMS (micro-electro-mechanical-systems) and NEMS (nanowire) versions of SI sensors employing the same kinds of emitter materials provide significantly higher sensitivity, however, at the expense of a reduced chemical selectivity. In this paper, it is argued that such sensitivity-selectivity trade-offs arise from unselective physical ionization phenomena that occur in the high-field regions immediately adjacent to the surfaces of sharply curved MEMS (NEMS) emitter and collector electrodes.

## 1. Introduction

Surface ionization (SI) is a form of gas detection, which relies on the chemisorption of analyte molecules on heated solid surfaces and on the extraction of the formed analyte ions from the adsorbent surface towards an oppositely biased counter electrode. A very interesting feature of the SI process is that it is largely insensitive to small, high-ionization-energy molecules, like N_2_, O_2_, H_2_O, CO_2_, and methane, but very sensitive towards low-ionization-energy analytes, such as secondary and tertiary amines [1,2,3,4,5,6,7]. This kind of selectivity makes SI detection interesting for the detection of organic decay products, illicit drugs, and a range of other hazardous materials [8,9,10].

While much of the pioneering work on SI gas detection had been performed on macroscopic versions of noble and refractory metal ion emitters [1,2,3,4], more recent work concentrated on miniaturized kinds of SI devices, including MEMS and NEMS versions [11,12,13]. In these latter devices, different kinds of electrode arrangements were used to read out SI signals from noble-metal and metal oxide (MOX) emitters. An important result of this work was that the sensitivity and selectivity of the SI response is not only determined by the stoichiometry and nanomorphology of the emitter materials, but also to a large extent by the shape of those electrode configurations that are used to enable surface ionization readout. Particularly, it was observed that different electrode configurations can give rise to markedly different cross sensitivity profiles and to different sensitivity-selectivity trade-offs.

In the present paper, we aim to shed more light on those processes that underlie these different sensitivity-selectivity characteristics. To provide a background for our discussion, the most relevant results of our previous work are briefly reviewed in Section 2, Section 3 and Section 4. In Section 5, we then turn to the electrical field distributions in the different kinds of SI devices. There, we show that MEMS-miniaturized and nanowire devices contain high-field regions around the emitter and collector electrodes, which can support various forms of high-field ionization phenomena. In Section 6, we discuss these competing ionization processes and show that the primary and chemically selective SI signals may considerably be amplified by secondary physical ionization processes, albeit at the expense of a reduced chemical selectivity. Overall, our discussion shows that there is a wealth of high-field processes, which can modify the primary SI processes occurring at the surfaces of MEMS and NEMS versions of SI detectors, and that these call for further systematic experimental assessment and verification.

## 2. Surface Ionization: Principle Sensing Mechanism

**Figure 1:**
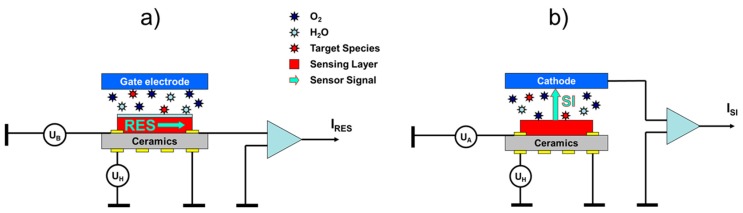
Metal oxide (MOX) gas sensor operated (**a**) in the conventional resistive (RES) readout mode; and (**b**) in the innovative surface ionization (SI) mode. In the RES mode, gas adsorption is monitored via changes in the in-plane resistivity of the MOX sensing layers. The suspended gate electrode is not normally present but may be used to modify gas adsorption processes via the electro-adsorption effect [16,17,18]. In the SI readout mode, changes in gas adsorption are monitored by observing flows of positive ions crossing the thin air gap (*d*_air_ ~0.1–1 mm) in between the heated MOX layer and the negatively biased counter electrode.

Surface ionization gas detectors show some resemblance to the more conventional metal oxide (MOX) gas sensors. This fact is shown in Figure 1, where we have schematically drawn the architectures of a conventional MOX gas sensor with a resistive (RES) readout (Figure 1a) and a surface ionization (SI) gas sensor with a vertical SI current readout (Figure 1b). Both kinds of sensors rely on functional materials deposited on heatable substrates. In RES gas sensors, the back-surface heater heats the MOX sensing layer on the front side to its optimum working temperature to induce gas surface interactions. While oxidizing gases, such as O_2_, NO_2_, and O_3_, withdraw electrons from n-type MOX materials reducing ones consume negative oxygen ion adsorbates, thereby forming neutral reaction products, such as H_2_O and CO_2_, and returning electrons to the MOX adsorbent layers [14,15]. The resulting resistivity changes are detected by external electronic circuits. In Figure 1a, we have additionally drawn an optional gate electrode, positioned above an air gap on top of the MOX sensing layer. Bias voltages applied to this electrode can generate electrical fields acting vertically to the electrical current flow inside the MOX layer. Such fields can influence both the charge transport inside the MOX sensing layer itself as well as the rates of charge exchange between the adsorbates and the sub-surface MOX layer. In this way, electro-adsorption and -desorption phenomena can be utilized to influence the sensitivity-selectivity characteristics of MOX gas sensing layers [16,17]. Inducing such effects requires surface electrical fields of more than $1\times 10^{5}\;V/\mathrm{cm}$ and small air gaps in the order of 1 μm or below, if battery-compatible low-voltage operation is desired. As generating such small air gaps requires methods of multi-wafer micro-assembly, this latter option is not usually followed [18].

Although architecturally very similar, surface ionization (SI) gas sensors follow a very different kind of operation principle as illustrated in Figure 1b. As described in more detail in our previous papers [5,6,7,8,9,10,11,12,13], surface ionization can be observed when molecules become absorbed at heated solid surfaces, and when valence electrons are raised from the highest occupied molecular orbitals (HOMO) of the adsorbates to one of the lowest unoccupied electron orbitals (LUMO) inside the adsorbent solid. In this way, positive ion adsorbates are formed, which remain bound to the adsorbent surface with some energy, $E_{\mathrm{ads}}$, until these become thermally released from the heated emitter electrode. Once liberated from the surface, the positive ions start to move along the electrical field lines stretching across the air gap between emitter and collector electrodes and rapidly attain a constant drift velocity, which results in an ion current, $I_{\mathrm{SI}}$, that can be detected in an external circuit. Another important difference relative to conventional MOX gas sensors is that SI gas detectors can, in principle, be realized using simple metal electrodes as ion emitters. In the early work on SI gas detection, both noble-metal and refractory-metal electrodes were investigated [1,2,3,4]. As refractory metals tend to get oxidized upon high-temperature operation in air, the actual ion emission in most cases is likely to have occurred from some sort of MOX material, which moves SI gas detectors somewhat closer to conventional MOX gas sensors.

**Figure 2:**
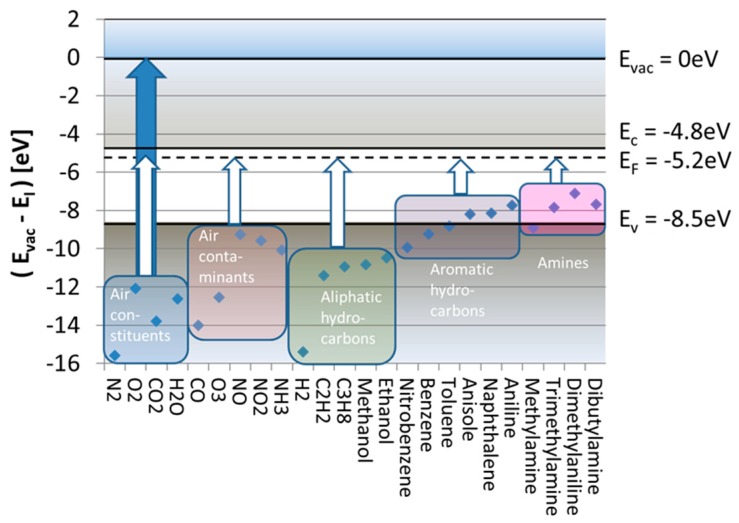
Free-space ionization energies, $E_{I}$, of selected molecules relative to the vacuum energy, $E_{\mathrm{vac}}$ (data redrawn from [19]). Blue arrows denote energy inputs for free-space ionization. White arrows denote energy inputs necessary to induce ionization after the molecules have become adsorbed on a SnO_2_ emitter surface. Colored rectangles denote different groups of analytes.

Figure 2 considers the subject of SI gas detection from its energetic point of view. Drawn there are the free-space ionization energies, $E_{I}$, of a range of different molecules relative to the vacuum energy level at which valence electrons become unbound, having zero kinetic energy ($E_{\mathrm{vac}}=0\;\mathrm{eV}$). This plot reveals that free-space ionization energies range between 7–16 eV, which means that, if direct ionization with photons is desired, ionization requires illumination with deep ultraviolet light. 

Much less energy is required in case these same molecules become adsorbed at heated solid surfaces. In case the emitter surface is a noble metal, such as, for instance, platinum (Pt), the LUMO energy is the metal Fermi energy, which lies roughly 5.7 eV below the vacuum energy [19]:

(1) $$E_{F,\mathrm{Pt}}=E_{\mathrm{vac}}-q\varphi_{\mathrm{Pt}}=-5.7\;\mathrm{eV}.$$

Ionization of adsorbed molecules, therefore, is energetically much more favorable than the ionization of free molecules. In case the emitter material is an n-type metal oxide, the majority of accessible LUMO levels are those empty electron states immediately above the conduction band edge ($E_{c}$). Alternatively, in p-type oxides, the accessible LUMO states will be those empty band states below the valence band edge ($E_{v}$). Considering the realistic case that nanocrystalline MOX materials will contain a multitude of localized defect states in the bandgap, which will pin the Fermi energy, the relevant LUMO energy levels will again coincide with the Fermi energy of the respective MOX materials. From these considerations, it is evident that surface ionization energies, $E_{\mathrm{SI}}$, can be controlled to some extent by a proper choice of emitter materials, emitter morphologies, and doping conditions.

To see how the energy expense for surface ionization, $E_{I}-E_{F}$, varies with the kind of analyte gas species, we consider in Figure 2 the special case of a SnO_2_ emitter electrode. Drawn there on the energy scale are horizontal lines representing the values of $E_{c},\;E_{v}$, and $E_{F}$ relevant to SnO_2_. The white arrows ending at $E_{F}$ indicate those energy inputs, $E_{I}-E_{F}$, that are necessary to ionize five relevant groups of analyte molecules. These arrows indicate that, even after adsorption, the ionization of all major background air constituents (N_2_, O_2_, CO_2_, H_2_O) still requires huge energy inputs of more than 5 eV, which are virtually impossible to supply out of the thermal reservoir of a heated emitter electrode. A similar situation arises in the case of H_2_ and common aliphatic hydrocarbons. Much better chances for thermal ionization exist if the adsorbates come from the groups of aromatic hydrocarbons or amines. In these latter cases, the remaining energy inputs drop to ΔE≈1−2 eV, only.

A key advantage of SI processes is that their probability scales in an Arrhenius-type manner with the ionization energy, $E_{\mathrm{SI}}$. In quantitative terms, the ionization current density, $J_{\mathrm{SI}}$, is expected to scale with the absolute temperature, T, and the analyte partial pressure, $p_{A}$, as [6,7]:

(2) $$J_{\mathrm{SI}}\sim p_{A}\;exp\left[-\frac{E_{\mathrm{SI}}}{k_{B}T}\right]=p_{A}\;exp\left[-\frac{\left(E_{I}-E_{F}\right)+E_{\mathrm{ads}}}{k_{B}T}\right]$$

In this equation, $E_{F}$ is the Fermi energy inside the adsorbent solid, $E_{I}$ the free-space ionization energy of the adsorbate, and $E_{\mathrm{ads}}$ its binding energy on the emitter surface. Due to this exponential cut-off criterion, all stable air constituents, such as N_2_, O_2_, CO_2_, and H_2_O, should remain completely undetectable. Analytically interesting molecules, such as aromatic hydrocarbons and amines, however, can potentially be detected with a high degree of selectivity. Concerning these latter groups of analytes, we mention that many toxic industrial compounds (TIC) feature aromatic rings [20], while many organic decay products as well as medical and illicit drugs feature amine functional side groups on their hydrocarbon backbones [8,9,10]. SI gas sensors therefore hold the promise of forming simple, sensitive, and selective sensors for interesting groups of analytes.

Fuji et al. [3,4] have studied surface ionization processes by applying mass spectroscopic analysis to the emitted ions. In this work, they found that surface ionization mainly proceeds through three different reaction channels:

Direct ionization: → M^+^ (3a)

Dissociative ionization: → (M − H)^+^ (3b)

Associative ionization: → (M + H)^+^. (3c)

In the first process, already discussed above, an electron is simply transferred from the adsorbate M across the HOMO-LUMO gap to the emitter surface, leaving a positive ion adsorbate $\left(M^{+}\right)$ and an electron transferred to the emitter surface ($e^{-}{}_{\mathrm{LUMO}}$):

(4a) $$M\to M^{+}+e^{-}{}_{\mathrm{LUMO}}.$$

In the other two processes, hydrogen is involved in the ionization process. To enable ionization processes in which analyte ion adsorbates either contain smaller or larger numbers of covalently bonded hydrogen atoms than the neutral analytes themselves, sources and sinks for the transferred hydrogen need to exist on the emitter surface. Keeping this in mind, an example for a dissociative ionization process is:

(4b) $$\left(M-H\right)+O^{-}\to M^{+}+e^{-}{}_{\mathrm{LUMO}}+{\mathrm{OH}}^{-}$$

Here, (M−H) stands for an analyte molecule with at least one covalently bonded hydrogen atom; $O^{-}$ is a surface oxygen ion, and ${\mathrm{OH}}^{-}$ a surface hydroxyl group. Similarly, an associative ionization process can be represented by:

(4c) $$M+H_{2}O\to {\mathrm{MH}}^{+}+{\mathrm{OH}}^{-}$$

In this latter process, the analyte molecule has become associated with a proton that has been withdrawn from an $H_{2}O$ adsorbate. Process (4c) is particularly advantageous in case analyte molecules with exceptionally high proton affinity are involved. Proton affinity (PA) is hereby defined as the energy gained in the reaction:

(5) $$M+H^{+}\to {\mathrm{MH}}^{+}.$$

**Figure 3:**
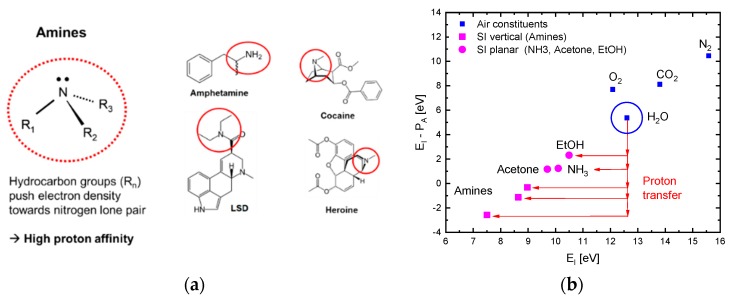
(**a**) Molecular structure of amines, leading to extremely high values of proton affinity (PA) and low effective ionization energies, $E_{I}-PA$; (**b**) residual ionization energy, $E_{I}-PA$, after proton attachment as a function of the free-space ionization energy, $E_{I}$, of different groups of analyte gases; Data redrawn from [19].

Figure 3a illustrates why high levels of proton affinity arise in the case of molecules with amine functional groups. Figure 3b further shows that molecules, M, with low free-space ionization energy, $E_{I}$, normally also feature large values of proton affinity [19]. This latter figure shows that, in the case of amines, the energy gain upon attaching a proton to a molecule M can exceed the energy input required for enabling free-space ionization of the compound ion ${\mathrm{MH}}^{+}$. This latter effect, in principle, turns the ionization of amines exothermic. The effect that ultimately turns the surface ionization of amines endothermic is that analytes first need to become chemisorbed before these can undergo surface ionization (Equation (2) and references [6,7]). Overall, the involvement of ${\mathrm{OH}}^{-}$ groups in reactions (4b) and (4c) shows that despite the large ionization energy of water molecules ($E_{I,H_{2}O}=12.6\;\mathrm{eV}$), the presence of water vapor in the ambient air is likely to impact the energetics of dissociative and associative ionization processes. Evidence into this latter direction is presented in Section 3.2.

## 3. Sensor Architectures and Sensing Materials

### 3.1. Vertical Read-Out Configurations

Whereas the early work on SI gas detection had been performed on macroscopic arrangements of noble and refractory metal emitters and collecting electrodes [1,2,3,4], we have employed in our own work MOX emitter materials commonly used in the field of resistive sensors, deposited as thin-films on ceramic substrates [21,22,23,24,25,26,27,28,29]. In this way, we have tried to make a whole range of technologies, which had been developed in the field of resistive MOX gas sensors, to become available in the much less investigated field of SI gas detection.

**Figure 4:**
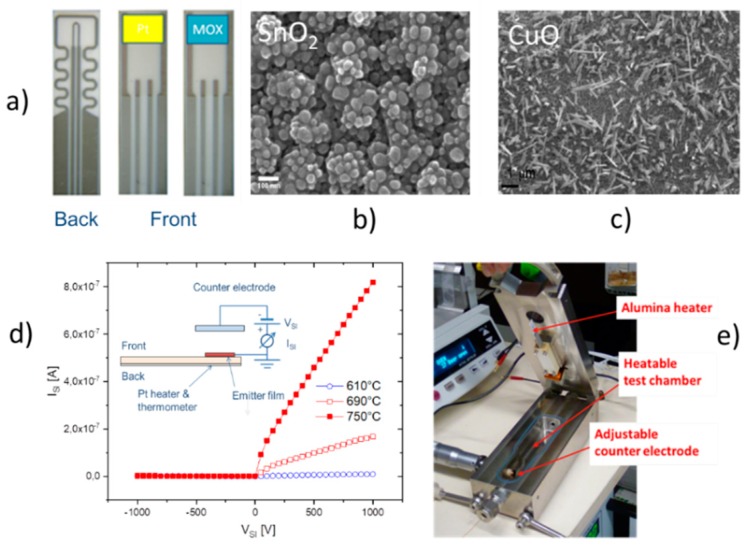
(**a**) Ceramic chips with back-surface Pt heaters and front-surface electrodes for contacting emitter layers; (**b**,**c**) MOX emitter materials deposited on the front-side using evaporation and thermal oxidation techniques [21,22,23,24,25]; (**d**) schematics of a vertical surface ionization read-out structure realized in a stainless-steel test chamber and resulting current-voltage characteristics observed upon ethene exposure (10^3^ ppm in synthetic air); (**e**) stainless steel measurement chamber featuring a heatable emitter chip (top lid) and adjustable counter electrode (lower left).

In a first stage, vertical readout architectures were investigated, which follow the scheme outlined in Figure 1b. Key building blocks were ceramic heater platforms with screen-printed Pt heater meanders on the rear-side and electrode patterns on the front-side surfaces as shown in Figure 4a. On top of these heater substrates, either thin-film Pt or various kinds of MOX materials could be deposited. In Figure 4b,c, we exemplarily show scanning-electron microscopy (SEM) images of SnO_2_ and CuO films, which had been formed by evaporation of Sn or Cu, respectively, and by subsequent thermal annealing in air [21,22,23,24,25]. As typical oxidation temperatures ($T_{\mathrm{ox}}\approx 600\;{}^{\circ}C$) are higher and lower than the respective metal melting points ($T_{m,\mathrm{Sn}}=232\;{}^{\circ}C$; $T_{m,\mathrm{Cu}}=1084\;{}^{\circ}C$), oxidation leads to MOX materials with very different nanomorphology (Figure 4b,c). Once deposited on the heater substrates, vertical readout structures were formed as schematically shown in Figure 4d. Also shown in this figure are current voltage characteristics that were observed upon exposing an SnO_2_ emitter film to ethene (C_2_H_4_) at the temperatures indicated. From the asymmetry of these diode-like characteristics it can be inferred that positive ions are extracted from the emitter surface. To avoid the complexities of multi-wafer micro-assembly, the heatable emitter chips on Figure 4a were mounted inside a custom-designed metal box with gas inlet and outlet ports as shown in Figure 4e. Upon mounting inside this box, electrical contacts to the emitter films and to the back-surface heaters could be made and a counter-electrode could be placed over the heated emitter films. Air gaps between emitter and collector electrodes with well-controlled widths could be formed with the help of a micro-meter screw that controlled the position of the counter electrode. Typically employed air gaps and bias voltages were $d_{\mathrm{air}}\approx 1\;\mathrm{mm}$ and $V_{\mathrm{SI}}=10^{3}$ V, respectively. Bias voltages to this counter electrode were applied through an isolated metal rod, running inside the micro-meter screw.

**Figure 5:**
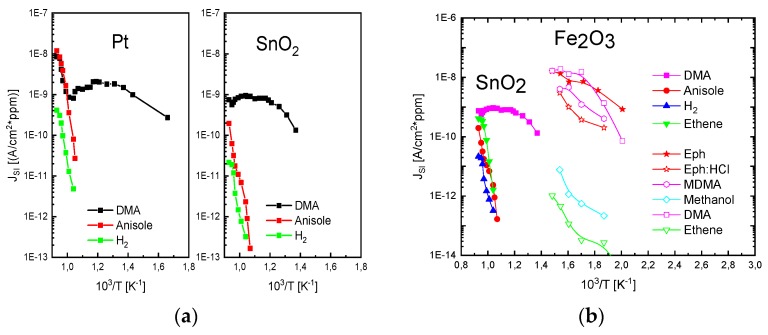
(**a**) SI ion current densities observed in vertical read-out structures as shown in Figure 4d. Emitters are thin-film Pt and SnO_2_ obtained by the thermal oxidation technique; (**b**) SI ion current densities observed from emitters consisting of thermally oxidized SnO_2_ and CVD-deposited Fe_2_O_3_ [8,9,10,26]. All materials show a very pronounced amine selectivity.

Figure 5 shows some key results obtained with this equipment. Figure 5a,b show that SI ion currents can be observed with both Pt noble-metal as well as with SnO_2_ emitters. Likely due to the higher work function of Pt ($q\varphi_{\mathrm{Pt}}\approx 5.7\;\mathrm{eV}$) and due to its higher catalytic activity, higher SI current densities could be obtained with Pt as compared to SnO_2_ emitters ($q\varphi_{\mathrm{SnO}2}\approx 5.2\;\mathrm{eV}$). A common property of both emitter materials is that very high levels of amine selectivity were observed when emitter temperatures were limited to temperatures lower than 600°C ($10^{3}/T\approx 1.15$). Figure 5b compares two kinds out of a larger variety of MOX emitter materials that were tested (SnO_2_, CuO, In_2_O_3_, MoO_3_, BaO, FeO*_x_*). Outstanding performance was obtained with CVD-deposited iron oxide emitters [8,9,10,26]. Unlike most other MOX materials, these generated sizeable ion current densities already at temperatures where conventional RES gas sensors are operated (250 °C<T<400 °C). With such films, parts-per-billion concentrations of various amines and illicit drugs could be detected [9]. Overall, the data in Figure 5 show that a proper choice of emitter materials can optimize the magnitude of SI currents and particularly the temperature range in which such currents can be observed. A common property of all three emitter materials is a very high level of amine selectivity. The likely reason for this unusual degree of selectivity is that the ionization of amines with their exceptionally high proton affinity is energetically more favorable than the ionization of most other competitor species (see Figure 3b). Aromatic hydrocarbons obviously benefit to a lesser degree from low values of ionization energy and high values of proton affinity. Additionally, aromatic hydrocarbons appear to be burdened by large chemisorption energies ($E_{\mathrm{ads}}$), which make them less detectable than amines [6,7].

### 3.2. Planar Read-Out Configurations

**Figure 6:**
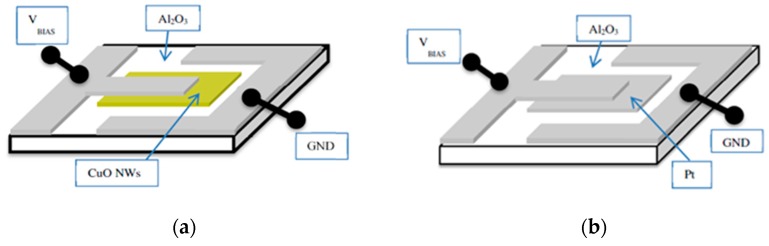
Planar SI sensor architectures built up on ceramic substrates with bottom-surface heaters: (**a**) Planar device featuring a CuO emitter layer (yellow) opposite to a Pt counter electrode (grey); (**b**) symmetrical reference device featuring both emitter and collector electrodes consisting of thin-film Pt. Reprinted from [28].

Attempting to overcome the problems of micro-assembly associated with the necessity of forming vertical read-out structures, the Brescia group investigated planar architectures of SI sensor devices as shown in Figure 6 [27,28,29]. Substrates were 2 × 2 mm^2^ alumina chips with back-surface Pt heater meanders and front-surface Pt electrodes for the SI readout. On top of the emitter electrode, either thin-film Pt or MOX films with controlled stoichiometry and nanomorphology could be deposited. Whereas all Pt structures were prepared by DC magnetron sputtering at 300 °C, copper oxide nanowires were prepared by room-temperature PVD deposition and thermal annealing in air afterwards [22,23,24,25]. Easy-to-use lithographic technologies based on shadow masks were used to pattern the device layout with a spatial resolution better than 100 µm. To assess the effects of intrinsic nanomorphology of the MOX emitter layers on the SI process, devices with MOX emitters and Pt collector electrodes (Figure 6a) were compared to symmetrical reference devices in which both emitter and collector electrodes consisted of thin-film Pt (Figure 6b).

Regarding the vertical readout configurations discussed above, two important differences stand out: Firstly, in the planar layout, both electrodes are heated, which means that both can potentially induce ionization; secondly, emitter and collector electrodes take the form of 1d-line emitters and line collectors facing each other across an insulator gap of approximately 200 μm. In detail, both 1d-electrodes feature lengths in the order of 1 mm in the planar and only a few tens to several hundred nanometers in the vertical direction. As we will see below, this 1d-line configuration strongly enhances the electrical field strengths acting at the emitter and collector surfaces to an extent that SI current emission can be observed at bias voltage and emitter temperature levels much lower than in the vertical readout configuration.

**Figure 7:**
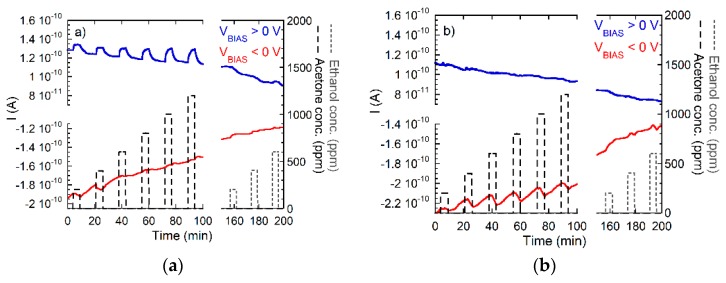
Response of CuO (**a**) and Pt emitters (**b**) to different concentrations of acetone and ethanol in dry air. Bias voltages were ±30 V applied to the central electrode (with reference to Figure 6) and substrates being heated to 350 °C.

Figure 7a summarizes sensor response data obtained on a device as shown in Figure 6a to acetone and ethanol both with the CuO electrode being positively biased (blue) and negatively biased (red). Turning to the case of positive bias on the CuO electrode first, it is seen that a SI response to acetone ($E_{I,\mathrm{Ac}}=9.7\;\mathrm{eV}$) can be observed, while ethanol (EtOH), with its higher ionization energy ($E_{I,\mathrm{EtOH}}=10.49\;\mathrm{eV}$*),* remains undetected. When the polarity is reversed, small current oscillations can be observed upon acetone exposure and no response at all upon ethanol exposure. As the weak current oscillations upon acetone exposure do not scale with the gas concentration, we interpret both results as evidence that negative ion emission from CuO nanorods does not occur. Figure 7b shows the results when the same experiment is repeated, this time, however, with the symmetrical reference device shown in Figure 6b. In this latter case, neither an acetone nor an ethanol response can be observed when the center electrode is positively biased. When negative bias is applied to the center electrode, no response is observed upon ethanol exposure and again small oscillations whose amplitudes do not scale with the acetone gas concentration. These negative results rule out any major emission of either positive or negative ions from thin-film Pt electrodes under the bias and temperature conditions applied to this kind of planar sensor layout. Overall, these first results are consistent with the point of view that positive ions are emitted from the CuO nanowires when these are exposed to low-ionization-energy gases, such as acetone, and that no emission occurs when the free-space ionization energy of the analyte gases is slightly higher than 10 eV. The higher emission from CuO relative to Pt can potentially be attributed to two reasons: (i) The intrinsic 3d-nanowire morphology of the CuO line-emitters, and/or (ii) a Fermi-level $E_{F,\mathrm{CuO}}$ lying deeper below $E_{\mathrm{vac}}$ than in Pt. A decision between both alternatives was not yet possible at the time of writing.

**Figure 8:**
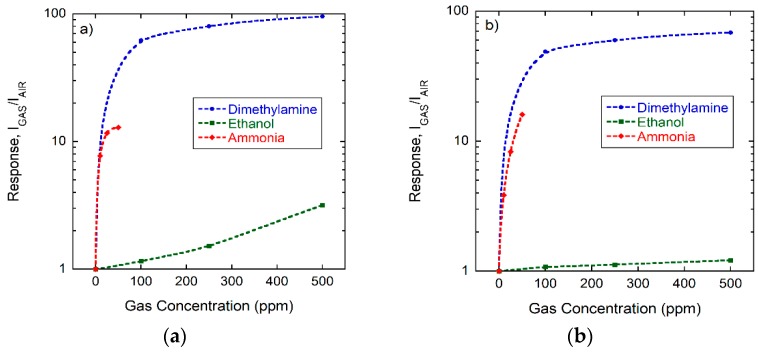
Response of CuO emitters to different concentrations of dimethylamine, ammonia, and ethanol applied in (**a**) dry and (**b**) humidified synthetic air (RH = 50% @ 20 °C). All measurements have been acquired with bias voltages of +200 V applied to the CuO electrode and with the sensor substrate heated to 400 °C.

That free-space ionization energy is the key determining detection criterion is further confirmed by the data summarized in Figure 8. In this latter experiment, a CuO device as shown in Figure 6a was exposed to dimethylamine, ammonia, and ethanol in different humidity backgrounds. While Figure 8a deals with exposures in backgrounds of dry synthetic air, Figure 8b shows the respective responses in backgrounds of humidified synthetic air (RH=50% @ 20 °C). The three compounds studied in this experiment feature ionization energies of $E_{I,C2H7N}=8.24\;\mathrm{eV}$, $E_{I,\mathrm{NH}3}=10.1\;\mathrm{eV}$, and $E_{I,\mathrm{EtOH}}=10.49\;\mathrm{eV}$; the respective proton affinities are $PA_{C2H7N}=9.63\;\mathrm{eV}$, $PA_{NH3}=8.85\;\mathrm{eV}$, and $PA_{\mathrm{EtOH}}=8.05\;\mathrm{eV}$. The data in Figure 8 demonstrate with even better clarity that the ionization response, $R_{SI}=I_{gas}/I_{air}$, increases with decreasing ionization energy and increasing proton affinity. A second relevant observation is that the response to all gases decreases as the humidity level in the background air is increased. EtOH, with the largest ionization energy, is thereby most affected. This latter result is understandable in view of the data presented in Figure 3b as a transfer of a proton from H_2_O to EtOH is energetically less favorable than in the case of NH_3_ and particularly in the case of dimethyl amine [27,28,29].

Summarizing our observations on the planar sensor layout, we find as a key result that a range of relevant gases can be ionized and detected at temperatures where conventional resistive MOX gas sensors usually work. In contrast to resistive MOX gas sensors, where combustibility is the key detection criterion, the SI response is more strongly determined by the free-space ionization energy and the proton affinity of the detected compounds. Additionally, although H_2_O is not directly detectable because of its high ionization energy ($E_{I,H2O}=12.6\;\mathrm{eV}$), it appears to influence intermediate steps in the SI process, which results in some cross sensitivity towards water vapor [28].

### 3.3. MEMS and NEMS Miniaturization Approaches

**Figure 9:**
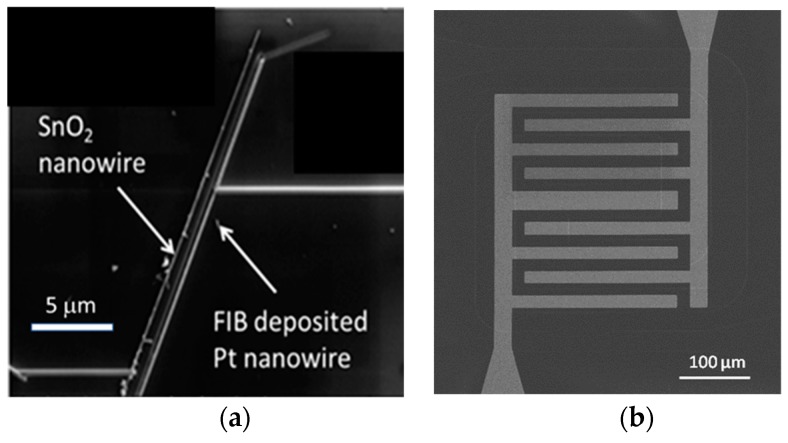
(**a**) Microscopy image of the surface of an oxidized silicon wafer with a pair of parallel nanowire-electrodes placed on top. Reproduced with permission from [11]. Copyright Royal Society of Chemistry, 2011; [13]. Copyright Elsevier, 2013; (**b**) Microscopy image of the surface of a MEMS micro-heater chip featuring a pair of interdigital Pt electrodes. Reproduced with permission from [12]. Copyright Elsevier, 2013.

Minimization of the heating power consumption using micro-miniaturization approaches has been a major driver of innovations in the field of MOX gas sensors with resistive readout. Key innovations were the introduction of MEMS microheaters [30,31,32] and the discovery of self-heating phenomena in single-nanowire devices [33,34,35,36,37]. In this way, the power consumption of conventional MOX gas sensors could be decreased from roughly 1W per device into the range of tens of milli-Watts with MEMS, and even into the micro-Watt range using self-powering nanowire devices. Both kinds of micro-miniaturization and heating approaches have also shown promise in the field of SI gas sensors. Attempts at arriving at such devices have been made by replacing the deposited thin-film emitter materials in the macroscopic arrangements of Figure 4d,e by silicon chips with pairs of parallel nanowires (Figure 9a) or by commercial MEMS microheaters with pairs of Pt interdigital electrodes, respectively (Figure 9b).

**Figure 10:**
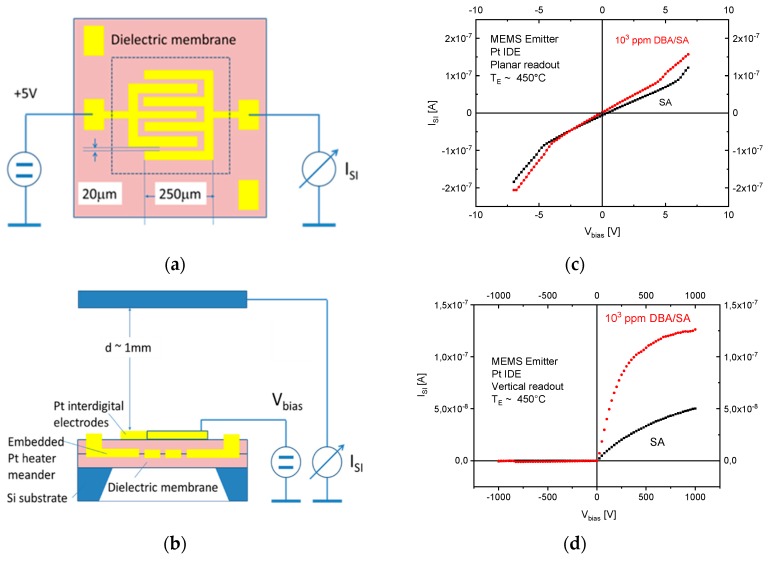
(**a**) Planar and (**b**) vertical readout of SI currents developing at the sharp, lithographically defined edges of the Pt interdigital electrodes of a MEMS microheater chip (Figure 9b). The dotted square in (**a**) denotes the extension of the free-standing membrane area. SI currents as a function of voltages applied across the interdigital Pt electrode gap (**c**), or across the air gap between the microheater and suspended counter electrode (**d**). Reproduced with permission from [12,13]. Copyright Elsevier, 2013.

Both kinds of devices can be operated in two principally different ways as schematically shown in Figure 10a–d. In the first mode of operation (Figure 10a), the ion extraction potential is applied between the pair of Pt electrodes. There, ion emission and ion collection occur between electrodes, which, essentially, face each other in the form of two parallel Pt nanowires. This first mode of operation, obviously, is very similar to the one used in the planar ceramic SI devices discussed above. The second architectural option is shown in Figure 10b. There, the chips are mounted on top of the ceramic heater substrates shown in Figure 4a and the chips with their heater substrates are placed into the metal chamber shown in Figure 4e, where a flat-plate counter electrode is placed over the microheater to realize a vertical readout structure. With these two biasing options, very different kinds of current-voltage-(IV)-characteristics can be observed as shown in Figure 10c,d. Whereas symmetrical characteristics are observed in the planar configuration (Figure 10c) both under synthetic air as well as under exposure to dibutyl-amine (DBA), characteristics with a very clear rectifier behavior are observed when vertical readout conditions are applied (Figure 10d). Also evident from these latter two figures is that background currents in synthetic air are smaller in the vertical as opposed to the planar readout case. Very similar results than with the MEMS microheaters were also observed with the kinds of parallel nanowire devices shown in Figure 9a, which shows that the ion emission is indeed coming from the sharp lithographically defined edges of the Pt interdigital electrodes [11,12,13].

## 4. Impact of Device Architecture on Sensitivity/Selectivity Characteristics

**Figure 11:**
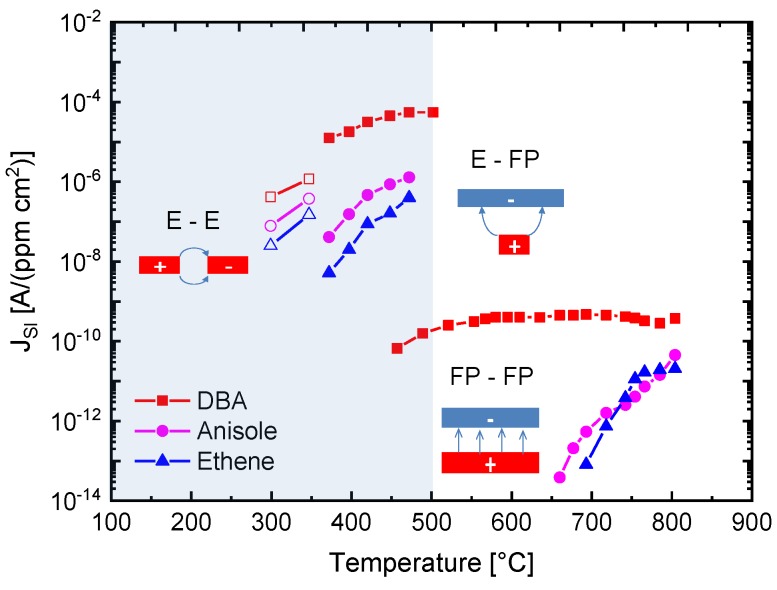
Temperature dependence of the surface ionization current density in dependence on analyte gas species and emitter and collector electrode arrangements (FP-FP: Flat-plate to flat-plate; E-FP: Edge to flat-plate; E-E: Edge to edge). All data in this figure were obtained with platinum emitter films. Heated emitter electrodes are marked in red with a positive sign on and cold collector electrodes in blue with a negative sign on. In E-E-type devices, both emitter and collector electrodes sit on a common heated substrate. The blue shading indicates the temperature range in which conventional MOX gas sensors are operated.

The results in Figure 10 demonstrate with clarity that very different kinds of IV characteristics can be observed when one and the same kind of emitter material is shaped into different readout geometries. Whereas in these MEMS devices, the readout structures take the form of edge-to-edge (E-E) or edge-to-flat plate (E-FP) geometries, the initially investigated vertical read-out structures, shown in Figure 4 and fitted with thin-film Pt electrodes, take the form of flat plate-to-flat plate (FP-FP) capacitor structures. In Figure 11, we compare the SI current densities generated by these three different kinds of device structures as functions of the emitter temperature and in response to three gases with different free-space ionization energies: Dibutyl-amine, $E_{I,\mathrm{DBA}}=7.83\;\mathrm{eV}$; anisole, $E_{I,\mathrm{Ai}}=8.2\;\mathrm{eV}$; and ethene, $E_{I,\mathrm{EtOH}}=10.51\;\mathrm{eV}$. The device configurations, on which the individual data have been obtained, are sketched in the form of insets in Figure 11. In comparing the different data sets, the most striking observation is that the obtainable ion current densities and the level of amine selectivity of the SI response are both critically dependent on the form of the ion-emitting and ion-collecting electrodes [6,7,12,13].

By far, the lowest ion current densities are produced by FP-FP configurations, in which both emitter and collector electrodes take the form of flat-plates [5,6,7]. In these parallel-plate capacitor-type devices, emitters and collectors are separated by an air gap of 1mm, and a DC potential of 1000 V is applied between both electrodes to allow ion currents to flow through the air gap. In this first vertical readout configuration, sizeable ion current densities could only be observed at moderate emitter temperatures, in the case of dibutyl-amine (DBA), which features a particularly low ionization energy ($E_{I,\mathrm{DBA}}=7.83\;\mathrm{eV}$) and a concomitantly high proton affinity of $PA_{DBA}=9.5\;\mathrm{eV}$. Analytes with slightly higher ionization energies ($E_{I,\mathrm{Ai}}=8.2\;\mathrm{eV}$, $E_{I,\mathrm{EtOH}}=10.51\;\mathrm{eV}$) hardly yielded any observable ion currents at emitter temperatures lower than 600 °C. FP-FP configurations, therefore, are almost selective to low-ionization-energy amines with a concomitantly high proton affinity. From an application point of view, it is important to note that a whole range of illicit drugs does fall into this category. FP-FP types of sensors therefore can make very simple, sensitive, and selective sensors for illicit drugs [8,9,10].

This situation changes completely when emitter electrodes with radii of curvature in the order of several tens of nanometers are employed. As described above, such emitters can be realized either by employing single nanowires [11,13] or by using the sharp, photo-lithographically defined edges of Pt interdigital capacitors as line emitters [12]. In case these line emitters are read out with the help of flat-plate collector electrodes as before, orders of magnitude larger ion current densities can be obtained at significantly lower emission temperatures ($T_{e}\approx 300-500\;{}^{\circ}C$). As evidenced in Figure 11, an obvious downside of the higher ionization current densities and the lower emission temperatures is a significantly lower amine selectivity of the SI response.

A further lowering of the emitter temperatures can be attained by shaping both emitter and collector electrodes into the forms of line emitters and line-collectors, respectively [11,12,13]. In these kinds of devices, both emitter and collector electrodes sit on a common insulating substrate while being heated to a common emitter and collector temperature, *T*. The data in Figure 11 show that with such pairs of line-emitting and line-collecting devices, emission temperatures as low as 200 °C can be attained; however, at the expense of an almost complete loss of amine selectivity.

**Figure 12:**
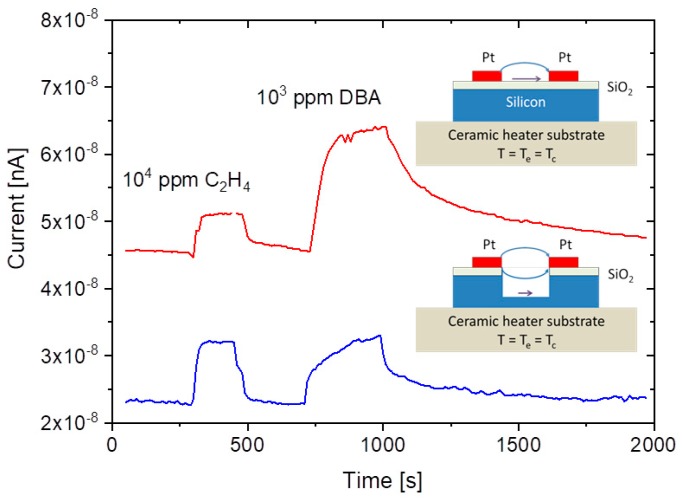
Gas response of an edge-to-edge SI detector configuration towards 10^4^ ppm of ethene and 10^3^ ppm dibutyl-amine (DBA) admixed to synthetic air. Top panel: Conventional edge-to edge device; bottom device with additional trench saw cut to suppress parasitic surface currents.

A common problem of all presently investigated E-E readout structures is that these have been realized on common heater substrates. Consequently, these devices contain heated collector electrodes, which make them somewhat difficult to compare to the FP-FP and E-FP devices discussed above. A first problem is that heated collector electrodes, in principle, could emit negative ions, which would raise the positive ion currents emerging from the hot, positive emitter electrodes. A second problem is that the presently available E-E devices do not contain a fully developed air gap in between the emitter and collector electrode. Parasitic surface currents creeping across the insulator gap between emitter and collector electrodes could therefore superimpose on the ion currents flowing through the air gap in the upper half-sphere of the device. Attempting to assess the magnitude of such parasitic current flows, Figure 12 shows data obtained on E-E devices both with and without a deep saw-cut trench in between emitter and collector electrodes to suppress any parasitic surface currents. This comparison shows that a slightly better DBA/ethene selectivity is obtained when a deep trench cut had been formed. This enhanced selectivity, however, is orders of magnitude smaller than in the other device configurations considered above. In view of this situation, it is unlikely that the selectivity characteristics of E-E devices are seriously limited by parasitic surface currents. Rather, the different electric field profiles across the readout gap seem to play a dominating role.

## 5. Electrostatic Potential and Field Distributions

As the huge enhancements of the ionization efficiency in nanowire or edge-emitting SI devices are undoubtedly related to the enhanced electrical field strengths that exist at the surfaces of nanowire or MEMS edge emitters, estimates for the acting field strengths are required. Detailed potential and electrical field distributions can be obtained from finite-element modelling (FEM). As, in this paper, we are more concerned with the gross features of the different electrode configurations, we prefer here to use analytical approximations for the acting field strengths. The advantage of analytical solutions is that these provide a much clearer view on those geometrical and electrical parameters that determine the actual device behavior.

**Figure 13:**
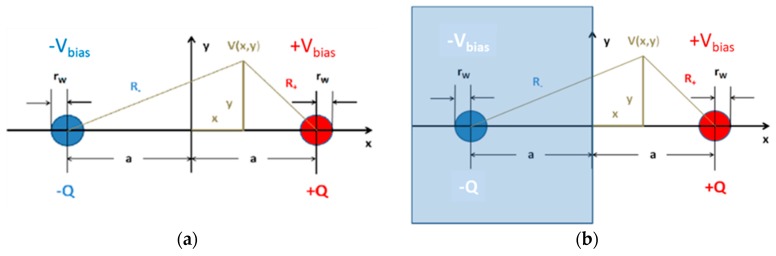
(**a**) Charge and electrical potential distribution in a parallel-nanowire arrangement; (**b**) charge and potential distribution with a positively charged nanowire placed in front of an electrically conducting medium, filling the half-space, *x* < 0. The negative charge distribution in this case is the image charge induced by the positively charged emitter wire.

The idealized device configurations, which we should like to analyze in more detail, are shown in Figure 13. Figure 13a presents a cross section through a parallel-nanowire arrangement with both nanowires being separated by a distance, 2a. The radius of curvature of both nanowires is assumed to be $r_{W}\ll a$. Figure 13b shows this same double-wire arrangement. This time, however, the negatively charged nanowire represents the image charge that is induced, when a positively charged nanowire is placed opposite to an electrically conducting medium filling the half-space, x<0. The induced image charge yields the same solution for the potential, V(x,y), as before, however, with the validity of V(x,y) being limited to the half-space, x≥0. In this latter case, the potential, V(0,y), yields the potential distribution on an electrically conducting flat counter electrode placed in the plane, x=0. In summary, the two idealized double-line arrangements can provide analytical approximations to the electrical potential and field distributions both in a parallel nanowire arrangement [11] and in a nanowire/flat-plate arrangement [13]. Both arrangements also provide reasonable estimates for the acting field strengths when the nanowire emitters and collectors are replaced by pairs of interdigitated electrodes with sharp, photo-lithographically defined edges [12].

The potential distribution, V(x,y), generated by both nanowires at the position, (x,y), can be obtained by superimposing the textbook solutions for the potential of a single electrically charged infinite wire [38,39], yielding:

(6) $$V\left(x,y\right)=\frac{V_{\mathrm{bias}}}{\ln\left[\frac{r_{W}}{2a}\right]}\;ln\left[\frac{\sqrt{{\left(a-x\right)}^{2}+y^{2}}}{\sqrt{{\left(a+x\right)}^{2}+y^{2}}}\right]$$

While V(x,y) vanishes in the symmetry plane, i.e., V(0,y)=0, V(x,y) attains the values, $V\left(a,0\right)=V_{\mathrm{bias}}$ and $V\left(-a,0\right)=-V_{\mathrm{bias}}$, at the nanowire positions.

Considering the symmetries of the parallel and vertical nanowire arrangements, it is sufficient to concentrate on the line of sight between the nanowire emitter and the real or virtual collector wires:

(7) $$V\left(x\right)=\frac{V_{\mathrm{bias}}}{ln\left[\frac{2a}{r_{W}}\right]}\;ln\left[\frac{\sqrt{{\left(a+x\right)}^{2}}}{\sqrt{{\left(a-x\right)}^{2}}}\right]$$

and:

(8) $$E_{x}\left(x\right)=\frac{V_{\mathrm{bias}}}{ln\left[\frac{2a}{r_{W}}\right]}\;ln\left[\frac{2a}{a^{2}-x^{2}}\right]$$

**Figure 14:**
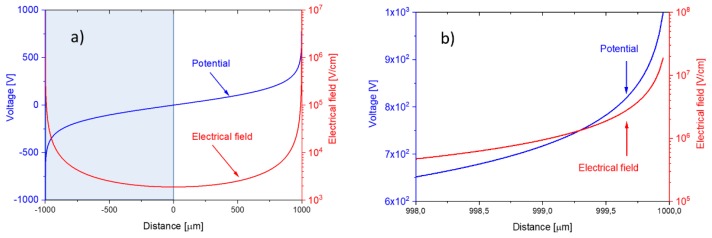
(**a**) Potential (blue) and electrical field distribution (red) inside the air gap separating a pair of parallel nanowires. The right-hand part of both distributions (*x* > 0) represents the potential and electrical field distributions of a positively charged nanowire facing a conducting flat-plate collector electrode placed in the plane, x=0; (**b**) potential and electrical field distributions close to the nanowire surface.

Both distributions are drawn up in Figure 14, assuming an external bias voltage of $10^{3}\;V$ and a nanowire-to-nanowire distance of 2a=2 mm. The nanowire diameter, $2r_{W}$, in this case was assumed to be 50 nm. Concentrating on the right-hand side of both distributions, i.e., at the half-space, x≥0, the truncated distributions also represent the case of a positively charged nanowire facing a flat-plate collector electrode, placed at a distance of a=1 mm.

The data in Figure 14a,b show that field strengths in the order of $10^{7}\;V/\mathrm{cm}$ are generated directly at the nanowire surface. Fields higher than 10^5^ V/cm reach out to distances of about 5 μm from the nanowire surface. In the middle, in between both nanowires, the field strength drops below the average parallel flat-plate capacitor value of $E_{av}=V_{\mathrm{bias}}/a=10^{4}V/\mathrm{cm}$. Evaluation of Equation (7) at the positions x=0 (Equation (9a)) and at the nanowire surface itself (Equation (9b)) shows that the electrical field at a flat-plate collector electrode is sensitive to the nanowire-collector distance, a, whereas the field at the nanowire surface itself is largely determined by the nanowire radius of curvature, $r_{W}$:

(9a) $$E_{\min}=E\left(x=0\right)=\frac{V_{\mathrm{bias}}}{\frac{a}{2}\;ln\left[\frac{2a}{r_{W}}\right]}$$

(9b) $$E_{W}=E\left(a-r_{W}\right)=\frac{V_{\mathrm{bias}}}{r_{W}\;ln\left[\frac{2a}{r_{W}}\right]}$$

## 6. Electrode Processes

### 6.1. Parallel Flat-Plate Geometry

**Figure 15:**
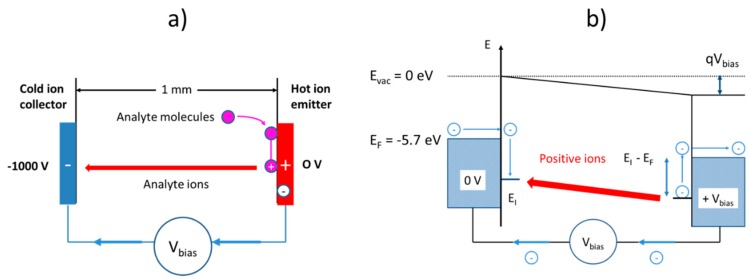
Surface ionization using a parallel flat-plate configuration: (**a**) Geometric electrode arrangement; (**b**) energetics of ion generation, ion transport, and ion collection.

The simplest and most obvious approach towards surface ionization is employing a parallel flat-plate electrode configuration as shown in Figure 15a. There, a Pt emitter film is heated to temperatures in the 500–700 °C range and placed opposite to a cold collector electrode positioned at a distance, d≈1 mm. Provided the lateral extensions, L, of both electrodes are large compared to the air gap width, (L≫d), a homogeneous electrical field of magnitude, $E_{\mathrm{av}}=V_{\mathrm{bias}}/d$, will emerge when a positive bias potential, $V_{\mathrm{bias}}$, is applied to the emitter electrode. In case analyte molecules adsorb on the hot emitter surface, valence electrons of the adsorbates may get thermally excited to the lowest-lying electron states inside the emitter electrode and turn into positively charged analyte ion adsorbates. Once thermally released from the emitter surface, the positive ions travel toward the cold collector electrode where they discharge again, reforming neutral analyte molecules. In this way an electrical current is generated in the external electrical circuit as illustrated in Figure 15b.

As revealed from Figure 11, the attractivity of this parallel-plate capacitor geometry is its extreme level of amine selectivity. The key reason for this selectivity is that the ionization is completely confined to the chemically selective surface ionization processes occurring at the hot emitter electrode and that any other physical ionization processes that might occur during the ion transport to the negative counter electrode are strictly ruled out. This latter fact is revealed if one follows the travel of an analyte ion, that has become surface-ionized at the positive emitter electrode, toward the negative counter electrode. During its travel through the air gap, such an analyte ion will suffer frequent gas-kinetic collisions. In such collisions, it can lose all or parts of its kinetic energy that it had gained upon accelerating through empty space prior to its last collision. With the mean free path in ambient air being in the order of 0.3 μm and the average electrical field, $E_{\mathrm{av}}\approx 10^{4}\;V/\mathrm{cm}$, a travelling analyte ion does not gain, on average, more than 0.3 eV of kinetic energy in between gas-kinetic collisions, which is much lower than the ionization energies of all major air constituents and also any other kinds of analyte ions that may abound there. Secondary ionization events, therefore, cannot occur inside the air gap. Further, as the average ion energy is also significantly smaller than the electron work function of typical collector electrodes (φ=4−5 eV), secondary electrons also cannot be released upon ion impact on the collector electrode. For both reasons, the available ion current is limited to those ions that had become thermally ionized on the emitter electrode. The sensitivity and selectivity of a flat-plate electrode arrangement is therefore limited to the intrinsic sensitivity and selectivity of the SI process.

Numerical estimates show that using high-work function emitters ($\varphi_{\mathrm{Pt}}\approx 5.7\;\mathrm{eV}$, $\varphi_{\mathrm{SnO}2}\approx 5.2\;\mathrm{eV}$) and emitter temperatures in the order of 500 °C, only analytes with very low ionization energies, such as, for instance, dibutyl-amine (DBA, $E_{I,\mathrm{DBE}=7.83\;\mathrm{eV}}$), should get thermally ionized, while all others with ionization energies larger than about 10 eV should remain un-ionized. This is particularly true for the main constituents of the background air, i.e., N_2_; O_2_, CO_2_, H_2_O ($E_{I,N2}=15.58\;\mathrm{eV}$, $E_{I,O2}=12.07\;\mathrm{eV}$, $E_{I,\mathrm{CO}2}=13.78\;\mathrm{eV}$, $E_{I,H2O}=12.6\;\mathrm{eV}$) [19].

Regarding selectivity, a final matter of concern could be the nanomorphology of the emitter materials. As illustrated in Figure 4b,c, state of the art MOX materials exhibit granularity in the range of a few nm up to about 100 nm. The straight electrical field lines emerging from a flat counter electrode will therefore become distorted in the immediate neighborhood of the positive ion emitters. Elementary electrostatic reasoning [38], however, confirms that the two-dimensional (2d) forms of nanomorphology of the kinds shown in Figure 4b,c can distort the average homogeneous field inside the air gap only across a length scale comparable to the length scale of the nanomorphology itself. As this length scale is smaller than the average mean-free path in the adjacent gas phase, and even smaller than the lateral sizes of the 2d-emitter and collector electrodes, such morphological irregularity is unlikely to enable physical and therefore chemically unselective high-field ionization processes in the immediate vicinity of the ion emitting electrode surfaces. As we will see below, this situation changes completely in case emitting and collecting electrodes take the form of 1d-lines with linear extensions large compared to the size of the nanomorphology and the mean-free path in the gas ambient.

### 6.2. Nanowire-Flat Plate Configuration

**Figure 16:**
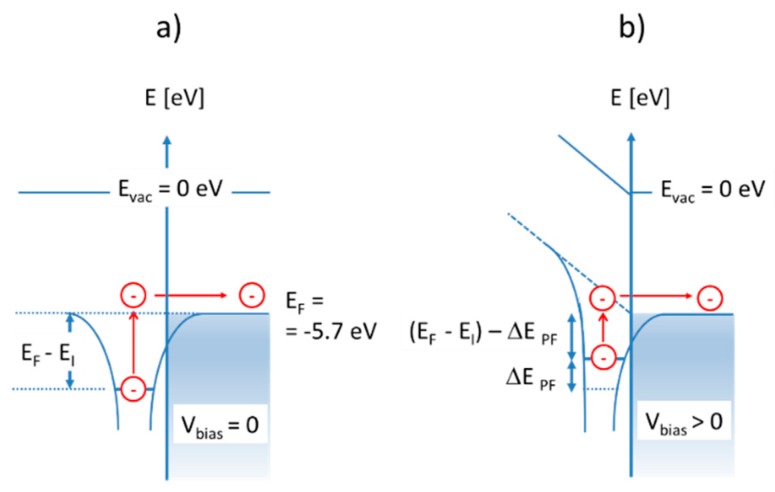
(**a**) Energetics of surface ionization under low-field conditions; (**b**) reduction of effective ionization energy under high-field conditions [40,41].

When the flat-plate emitter electrode is replaced by a nanowire or a pair of interdigital electrodes with sharp, photo-lithographically defined edges the ion emission is confined to a thin line with a very small radius of curvature. Along this line, electrical field crowding occurs, and much higher electrical fields can develop than on a flat-plate ion emitter. With electrical fields reaching values in the order of $10^{7}\;V/\mathrm{cm}$, voltage drops of a fraction of a Volt can develop over a single monolayer of adsorbates. When such sizeable voltage drops do occur over an adsorbate layer, the minimum excitation energy for surface ionization will be reduced by an amount, Δ*E*_PF_, as shown in Figure 16.

According to the Poole-Frenkel theory [40,41], the effective barrier reduction is:

(10) $$\Delta E_{\mathrm{PF}}=\sqrt{\frac{q^{3}E_{S}\;}{\gamma }}$$

In this latter equation, *E_s_* is the surface electrical field, *q* is the elementary charge, and *γ* is the effective dielectric constant at the adsorbate position. With *γ* taking on values in between the dielectric constants of free space and the bulk emitter material, values of barrier lowering in the order of 0.5 eV can be estimated when the acting field strengths are in the order of those given by Equation (9b). Generalizing Equation (2), one obtains for the SI current density emerging from a nanowire surface:

(11) $$J_{\mathrm{NW}}\;\sim \;E_{s}\;exp\left[-\frac{\left(E_{\mathrm{SI}}-E_{\mathrm{ads}}\right)}{k_{B}T}\right]\;exp\left[\frac{\sqrt{\frac{q^{3}E_{s}}{\gamma }}}{k_{B}T}\right]$$

Comparing this latter result to the current densities that can be drawn from a flat-plate emitter consisting of the same emitter material and heated to the same temperature, *T* (Equation (10)), huge emission enhancements, $J_{\mathrm{NW}}/J_{\mathrm{FP}}$, in the order of $10^{5}–10^{7}$ are inferred. A severe problem with such an interpretation is that these enhancements should be independent of the ionization energy of the analytes. In other words: Replacing a flat-plate emitter of any material by a nanowire of the same material, a pure Poole-Frenkel enhancement should yield large sensitivity enhancements without affecting the cross-sensitivity characteristics of the original flat-plate emitter. As this latter conclusion is evidently not supported by our observations (see Figure 11), other mechanisms of emission enhancement need to be considered.

Another phenomenon, which can occur at high voltages and at sharply curved surfaces, is the inception of corona discharges and the onset of charge multiplication processes within the discharge regions [42,43,44]. Corona discharges are normally a nuisance effect occurring in faulty high-voltage equipment. To get started, such discharges rely on extraneous inputs of energy, such as short-wavelength ultraviolet light, natural radioactivity, or cosmic rays. Once started, such discharges can get stationary, provided the initiation energy is replenished after all charges, generated by the initiating event, have been collected.

**Figure 17:**
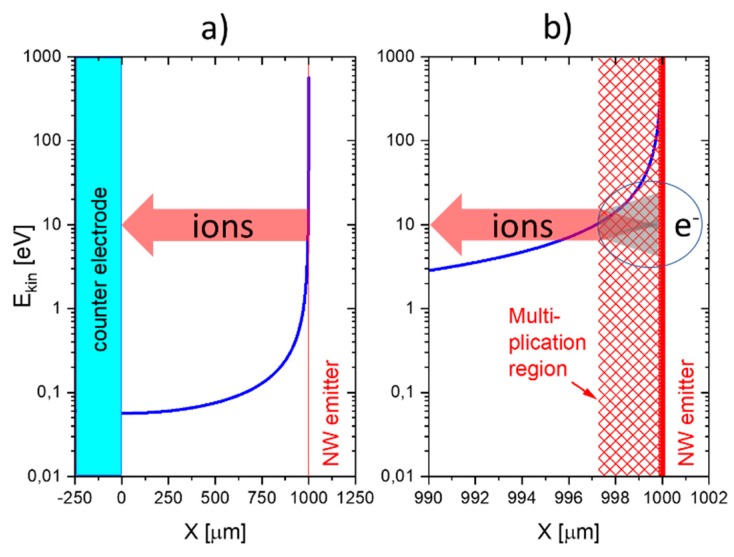
(**a**) Kinetic energy gained in between gas-kinetic collisions inside the electrode gap of an E-FP device (see Figure 11). The red arrow indicates the flow of positive ions toward the cold collector electrode through the low-field region where ions suffer gas-kinetic collisions but are unable to form secondary ion-electron pairs; (**b**) detailed view onto the air space immediately adjacent to the positive nanowire (NW) emitter. Inside this region, secondary ion-electron pairs are created, leading to a huge enhancement of the primary SI current.

In the SI gas sensors discussed here, the obvious initiating events are SI processes following gas adsorption at a hot and sharply curved emitter surface. Once generated, the primary positive ions are repelled from the emitter electrode towards the negatively charged collector electrode. Upon traversing the high-field region immediately adjacent to the emitter, each primary ion acquires enough kinetic energy in between gas-kinetic collisions to induce follow-on ionization events. In each of these events, a secondary ion and a free electron are generated. Such knock-on ionization events continue until the primary and secondary ions have travelled out to a distance where the acting electrical field strength has dropped below a level where the field can no longer re-supply the kinetic energy necessary to enable knock-on ionization. Figure 17 shows that such avalanching processes are confined to distances less than 3 μm from a typical nanowire surface, which corresponds to roughly 10 mean free paths at normal air pressure. Each primary ion, therefore, may generate up to $2^{10}\approx 10^{3}$ secondary ion/electron pairs. Once the travelling ions have left the high-field multiplication region, the positive ion cloud continues its travel towards the collector electrode in the form of a unipolar ion cloud until it becomes neutralized there. As upon arriving at the flat-plate counter electrode, the average ion energy has dropped to values in the order of 50 meV (see Figure 17a), i.e., to values much less than electron work functions of typical collector materials (Pt, Au, MOX, …), secondary electron emission is unlikely to occur there.

Recalling that in each of the $2^{10}\approx 10^{3}$ secondary ionization events not only a positive ion, but also a free electron has been created, the fate of these knock-on electrons still needs to be considered. Once generated, these electrons are firmly attracted towards the positively charged emitter electrode. As during their travel back, most electrons will acquire more than the free-space ionization energy of the background air constituents, additional knock-on ionization events will occur, which further enhances the amplification effect inside the multiplication region. Upon impact on the emitter electrode, any kinds of adsorbed gases may also become ionized in a chemically non-selective manner. In summary, it can be expected that up to $2^{10}\times 2^{10}\approx 10^{6}$ non-selectively generated electronic charges per initiating and chemically selective SI event can be generated. Returning to Figure 11, amplification factors in this order of magnitude are indeed observed when the response of E-FP and FP-FP devices to DBA vapors are compared.

Finally, recalling that the ion cloud impinging on the cold, flat-plate counter electrode is unlikely to knock out secondary electrons, which could travel back to the positively charged emitter electrode, the above-described electron and ion currents will be terminated after all secondary electrons and ions, which had been formed after the initiating SI event, have been collected. Enhanced diode currents in the outside circuits will therefore only be observed as long as analyte ions with low free-space ionization energies abound in the air gap between emitter and collector electrodes. Some finite background current, of course, will also be generated by background gases with higher ionization energies, by other extraneous events, and last-not-least by leakage currents across insulators inside the SI device.

### 6.3. Parallel Nanowire Configurations

**Figure 18:**
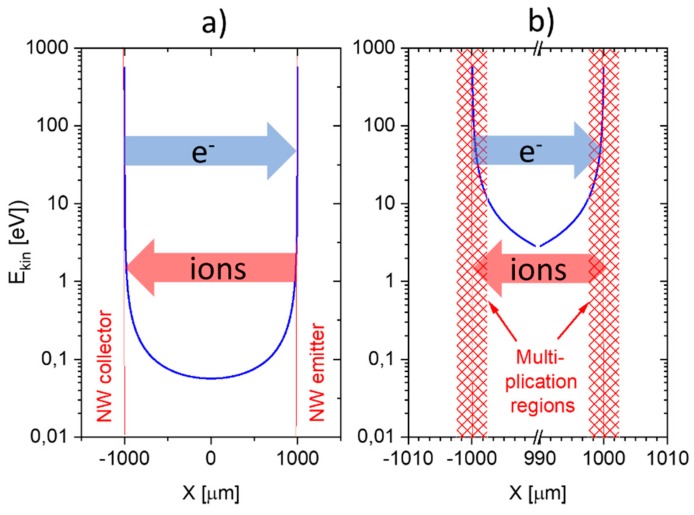
(**a**) Kinetic energy gained in between gas-kinetic collisions inside the electrode gap of an E-E device. The arrows indicate a flow of ions and a flow of electrons generated by positive ion impacts onto the collector electrode; (**b**) detailed view onto the air space immediately adjacent to the positive nanowire (NW) emitter and negative NW collector electrodes. The cross patterns indicate those regions in which positive ions emitted from the NW emitter can trigger avalanches of secondary ions.

In a parallel-nanowire configuration, the same enhancing mechanisms are operative in the immediate vicinity of the emitter surface as already discussed above (see Figure 18). Additional enhancing mechanisms, however, will occur as the positive ion showers impinge on a nanowire collector surface as opposed to a flat-plate collector electrode. With higher collector fields being available there (Equation (9b)), ions approaching the collector surface may now gain kinetic energies well above the electron work function of the collector electrode and may thus induce the release of secondary electrons from the collector nanowire without any form of thermal activation. This latter interpretation is again supported by our background current measurements on E-E devices. Unlike E-FP devices, which showed activation energies of about 3 eV, the background currents in our E-E devices only revealed activation energies of less than 0.3 eV. As explained above, the more numerous back-travelling electrons may induce large numbers of physical ionization events as they back-traverse in the two high-field regions in the vicinity of the collector and close to the positive emitter nanowire. Overall, and again in agreement with observations, more intense, less temperature dependent, and less chemically selective ionization currents are expected in E-E devices. Finally, as the electrical currents in the outside circuits return to their background values after all SI-initiating low-ionization-energy gases have been removed, it is evident that the secondary electron emission at the collecting nanowire electrodes does not fully replenish the charge flow that has been lost upon collecting the ion charge. Self-sustaining discharges, therefore, do not occur inside E-E devices.

## 7. Concluding Remarks

The efficiency with which adsorbed analyte gas species can become ionized at hot emitter surfaces depends both on adsorbate as well as on adsorbent parameters. Key adsorbate parameters are the free-space ionization energy, $E_{I}$, and the proton affinity, PA. These two quantities are constants of nature, which make the surface ionization of amines and aromatic compounds much more probable than the ionization of most other analytes. Key adsorbent characteristics are the electron work function of the emitter materials, $q\varphi_{\mathrm{em}}$, as well as the binding energies of the adsorbates, $E_{\mathrm{ads}}$, on the respective surfaces. These latter two parameters can clearly be influenced by the sensor developer. The considerations in this paper have shown that the magnitudes of SI current densities as well as the temperature ranges, in which these can be observed, depend to a large extent on a proper choice of the emitter material. In comparison to MOX materials, which are widely employed in resistively read out gas sensors, much less is known about those processes that control the efficiency of surface ionization processes. On the present kinds of emitter materials, the SI detection of aromatic compounds appears to be severely hampered by large adsorbate binding energies, $E_{\mathrm{ads}}$. In case such energies could be reduced by appropriate material design, sensitive and selective SI detectors for an additional interesting class of analytes would become possible.

Perhaps the most striking result of our investigations was that, besides the materials’ properties of the emitters themselves, the geometry of the ion emitting and ion collecting electrodes can have a huge impact on the sensitivity-selectivity characteristics of SI gas sensors. A key result in this direction was that the intrinsic amine-selectivity of SI emitters only shines up when both the ion emitting and ion collecting electrodes take the form of 2d-geometries with lateral extensions much larger than the nanomorphology of the emitter materials and the lengths of the mean-free paths in the adjacent air ambient (FP-FP devices). By shaping one and the same kind of material into the form of 1d-line emitters or line collectors, huge gains in sensitivity can be attained at the expense of worsening selectivity (E-FP and E-E devices). Our considerations suggest that such massive sensitivity-selectivity trade-offs occur due to the interference of unselective physical ionization processes with the initiating and chemically selective surface ionization processes. As such processes have received little attention in the field of gas sensors yet, significant progress appears to be possible in this area as well.

Our final remark concerns micro-miniaturized SI gas sensors, capable of working at low heating-power-consumption levels and at voltages compatible with battery-operated equipment. Here, our experiments on MEMS and NEMS versions of SI gas sensors have revealed that those micro-miniaturization technologies, which have already proven to be successful for arriving at resistive MOX gas sensors with very low heating power consumption, also appear to be applicable to arrive at low-power-consumption SI gas sensors. To fully arrive at the final goal of low-voltage operation, additional developments in the field of multi-wafer micro-assembly techniques are necessary. With the help of such assembly technologies it appears possible to produce small and rugged devices with air gaps in the range of micrometers, which will reduce the extraction voltages of vertically readout SI devices into the range of normal battery voltages.
